# Familial 46, XY Disorder of Sexual Development identified in a Ph+*BCR::ABL1^P210+^* Acute Lymphoblastic Leukemia septuagenarian female with *RCBTB2::LPAR6* fusion gene: a case report

**DOI:** 10.3389/fonc.2024.1339737

**Published:** 2024-07-18

**Authors:** Lingling Wang, Conglin Xi, Xinyu Zheng, Yongfen Huang, Hao Xu, Yuqing Miao, Yuexin Cheng

**Affiliations:** ^1^ Department of Hematology, The First People’s Hospital of Yancheng, The Yancheng Clinical College of Xuzhou Medical University, Yancheng, China; ^2^ Department of Oncology, The Second People’s Hospital of Huai’an, The Affiliated Huaian Hospital of Xuzhou Medical University, Huaian, China

**Keywords:** acute lymphoblastic leukemia, philadelphia chromosome, *BCR::ABL1*, 46, XY disorder of sexual development, familial

## Abstract

**Background:**

Familial 46, XY Disorder of Sexual Development (DSD) was discovered in a Ph+, *BCR::ABL1^P210+^* Acute Lymphoblastic Leukemia (ALL) female with *RCBTB2::LPAR6* fusion gene. Siblings developing 46, XY DSD are extremely rare. Patients with 46, XY DSD have much higher rates of gonadal cancers. Nevertheless, the incidence of hematologic malignancies in patients with DSDs has received little attention. *RCBTB2::LPAR6* is a rarely reported fusion gene in ALL.

**Case presentation:**

Herein, we report a rare case of a newly diagnosed Ph+, *BCR::ABL1^P210+^* ALL patient who was 77 years old and female by social sex. Whole Exome Sequencing (WES) and RNA sequencing revealed TET2 and NF1 mutations in addition to a rarely reported *RCBTB2::LPAR6* fusion gene and 17 other genes with uncertain clinical significance. The patient was surprisingly found to have a male karyotype. On ultrasound, neither the uterus nor the ovaries were discernible. A detailed family and marital history revealed that the patient had undergone surgery at an early age for an unexplained inguinal mass. She had slow pubertal development, scanty menstruation, and few overtly feminine characteristics. She had three marriages, but none succeeded in getting pregnant. The patient had never sought therapy for infertility due to the inaccessibility of medical treatment and a lack of medical knowledge. Her sister, 73 years old and female by social sex, who had amenorrhea in adolescence and was unable to conceive, had the same experience. To our surprise, she also had a male karyotype.

**Conclusions:**

Due to the absence of long-term social attention and follow-up, studies on the incidence of hematologic malignancies in patients with 46, XY DSD are incredibly uncommon. Siblings developing 46, XY DSD is extremely rare. We report the oldest patient diagnosed with 46, XY DSD. There have not yet been any reports of familial 46, XY DSD with a concurrent diagnosis of Ph+*BCR::ABL1^P210+^* ALL with a rarely reported *RCBTB2::LPAR6* fusion gene.

## Background

Ph+ ALL is rare in children, with an incidence of 2%-5%. However, it is the most common genetic subgroup in adult ALL (1), with an overall incidence of 20%-25%, and the incidence increases with age, with patients over 50 years of age accounting for more than 50%. Ph+*BCR::ABL1^P210+^* ALL is a rare type of ALL in the elderly. *RCBTB2::LPAR6* is a rarely reported fusion gene in Ph+*BCR::ABL1^P210+^* ALL.

DSD refers to a congenital chromosomal, gonadal, and phenotypic sex-linked developmental anomaly or mismatch that includes a variety of inborn metabolic anomalies and deformities, mostly marked by abnormalities of the external genitalia. There are three types of sex developmental anomalies: 46, XX DSD, 46, XY DSD, and sex chromosome DSD (2). Among these, 46, XY DSD has a karyotype of 46, XY, and may have a Müllerian duct structure. The external genitalia may be either normal male genitalia, ambiguous external genitalia, or even completely feminized (3). Familial 46, XY DSD is extremely rare, and studies of siblings with concurrent 46, XY DSD are uncommon, with only a few case reports to date. There are few studies on the prevalence of neoplasms, particularly hematologic malignancies, among DSD patients, especially the familial 46, XY DSD.

Herein, we report a rare case of a newly diagnosed Ph+*BCR::ABL1^P210+^* ALL patient who was 77 years female by social sex but had a male karyotype. A rarely reported *RCBTB2::LPAR6* fusion gene was discovered. After gathering a complete family history and marriage history, it was unexpectedly found that the patient’s sister who was 73 years was also a patient of 46, XY DSD.

Although both patients were in their seventies and no longer in need of childbearing, they were still puzzled about the cause of their infertility. With the consent of the patient and her family members, and the approval of the Medical Ethics Committee of The First People’s Hospital of Yancheng (Ethics Approval No. 2023-k-195), we initiated the study. Elderly patients with DSD do not receive much attention and support from society, partly due to the backwardness of medical testing technology in the past decades, and partly due to the poor accessibility of medicine for the general population. With the maturity of WES and RNA sequencing, more potential DSD patients may be diagnosed, and this population needs the attention of clinicians, not only limited to medical treatment but also more humanistic care and social support. This was our original intention in reporting this case.

## Case presentation

**Figure 1:**
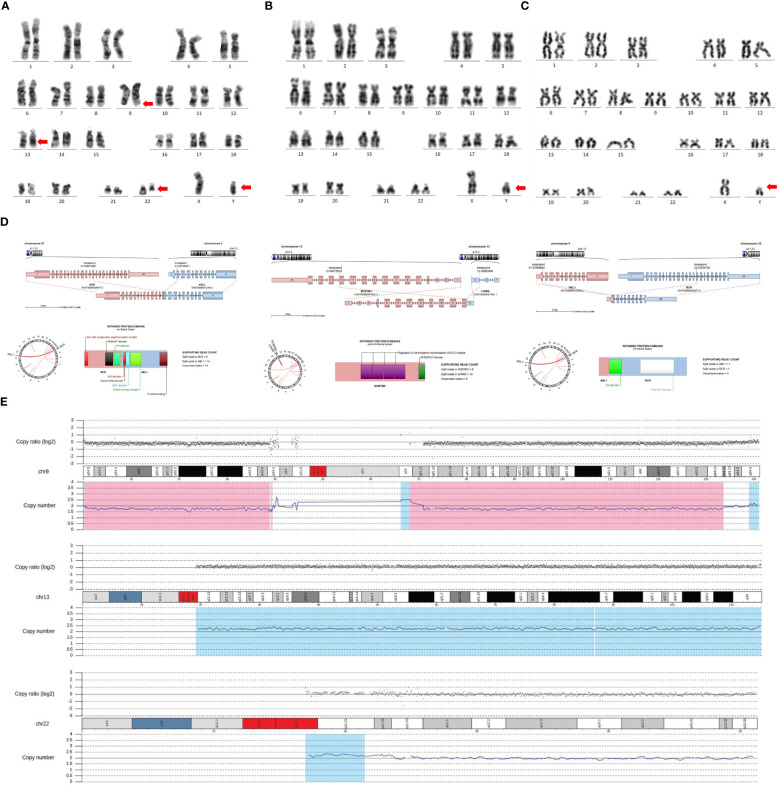
Testing techniques include G-banding technique, WES, and RNA sequencing. **(A)** G-banding analysis of bone marrow cells indicated the karyotype of 46, XY, t(9;22)(q34;q11.2)[10]/47, idem, +der(22)t(9;22)[1]/46, idem, del(13)(q12q14)[1]. **(B)** The chromosome analysis was performed with the patient’s peripheral blood, which still revealed a male karyotype. **(C)** The chromosomes of this patient’s sister were suggestive of a male karyotype. **(D)** WES and RNA sequencing indicated three fusion genes, including *BCR::ABL1*, *ABL1::BCR*, and *RCBTB2::LPAR6*. **(E)** WES and RNA sequencing indicated 46, XY DSD, with three additional chromosomal variants, del(9) (p24.3q34.12), dup(13) (q11q34), and dup(22) (q11.1q11.23). The genomic coordinates were chr9:g.190065_133643956del, chr13:g.19194200_115109878dup, and chr22:g.16962100_23627091dup, respectively.

In April 2023, a 77-year-old female presented to our hospital complaining of fatigue, bone pain, and skin petechiae. The patient had a history of coronary atherosclerotic heart disease, type 2 diabetes, and hypertension. A physical examination revealed no lymphadenopathy, hepatomegaly, or splenomegaly. She was found to have a WBC of 100 × 10^9^/L with 9% blasts, Hb of 9.1 g/dL, and platelet count of 14 × 10^9^/L. Serum aspartate aminotransferase was elevated to 145.0 U/L, albumin was 36.4 g/L, lactate dehydrogenase was elevated to 5999.0 U/L, and creatinine was 47.3 μmol/L. Serum electrolytes were normal. N-terminal brain natriuretic peptide was elevated to 881 pg/ml. The bone marrow biopsy revealed 28.5% blasts (CD13+, CD19+, CD20+, CD10+, CyCD79a+, CD34+, HLA-DR+). Fluorescence *in situ* hybridization (FISH) and reverse transcriptase polymerase chain reaction (RT-PCR) were positive for the presence of *BCR::ABL1* (coding for a 210-kDa protein). G-banding analysis of bone marrow cells indicated the karyotype 46, XY, t (9;22)(q34;q11.2)[10]/47, idem, +der(22)t (9;22)[1]/46, idem, del(13)(q12q14)[1] (**Figure 1A**).

The patient’s social sex was female, yet the chromosomes were of the male karyotype. We re-examined the patient’s peripheral blood for chromosomal verification, which was still suggestive of a male karyotype (**Figure 1B**). On ultrasound, neither the uterus nor the ovaries were discernible. A detailed family and marital history revealed that the patient had undergone surgery at an early age for an unexplained inguinal mass. She had slow pubertal development, scanty menstruation, and few overtly feminine characteristics. She had three previous marriages, but none succeeded in getting pregnant. The patient did not seek an early diagnosis and treatment because of medical inaccessibility when she was young and a lack of knowledge about medicine. Examination of the external genitalia revealed an enlarged clitoris. Laboratory analysis revealed only 5.47 (range: 10~30) pg/ml of estradiol, a significantly reduced quantity. Blood testing revealed that male testosterone levels were normal, at 0.2 (range: 0.1~0.75) ng/mL. In light of this, we speculate that this patient might be an unusual case of Ph+*BCR::ABL1^P210+^* ALL combined with 46, XY DSD. WES and RNA sequencing were performed to determine whether the patient had DSD-related mutations. NF1 [11.2%; NM_001042492: c.7194C>A(p.Tyr2398Ter)] and TET2 [39.9%; NM_001127208: c.3743T>C(p.Leu1248Pro)], as well as 17 other mutations, were detected (**Table 1**). In addition to the *BCR::ABL1* fusion gene, the *ABL1::BCR* fusion gene, and a novel *RCBTB2::LPAR6* fusion gene were also discovered (**Figure 1D**). Copy Number Variation Sequencing (CNV-Seq) confirmed that the patient’s sex chromosomes were XY, which was inconsistent with social gender, along with three autosomal variants (del(9) [p24.3q34.12), dup(13) (q11q34), and dup(22) (q11.1q11.23)] (**Figure 1E**). Her sister, a 73-year-old female by social sex, who had amenorrhea in adolescence and was unable to conceive, had the same experience. When we karyotyped the patient’s sister, we discovered, to our surprise, that she too had a male karyotype (**Figure 1C**). However, she did not suffer any gonadal or hematologic malignancies.

**Table 1:** Whole Exome Sequencing and RNA sequencing^a^ identified a total of 17 variants with undetermined clinical significance.

No	Mutant Gene	Chromosome Location	Nucleotide and Amino Acid Changes	Allelic Ratio
1	ARID1A (NM_006015)	chr1:27087947	c.2234G>A (p.Ser745Asn)	0.397
2	CHL1 (NM_006614)	chr3:391167	c.974G>A (p.Arg325His)	0.417
3	HACE1 (NM_020771)	chr6:105231979	c.1548G>A (p.Met516Ile)	0.456
4	ANKRD17 (NM_032217)	chr4:73963838	c.4973C>T (p.Thr1658Ile)	0.374
5	CYBA (NM_000101)	chr16:88717415	c.7C>T (p.Gln3Ter)	0.527
6	DNAH12 (NM_001366028)	chr3: 57391391	c.6565C>A (p.Gln2189Lys)	0.466
7	DNAH12 (NM_001366028)	chr3:57432425	c.3787–9T>C	0.541
8	FANCG (NM_004629)	chr9:35075597	c.1298G>A (p.Arg433Gln)	0.518
9	FAT2 (NM_001447)	chr5:150948447	c.46G>T (p.Ala16Ser)	0.595
10	HKDC1 (NM_025130)	chr10:71026409	c.2650C>G (p.Arg884Gly)	0.452
11	L4R (NM_000418)	chr16:27353547	c.176G>A (p.Arg59His)	0.507
12	NFATC4 (NM_001320043)	chr14:24845662	c.2408G>C (p.Gly803Ala)	0.519
13	PDE4DIP (NM_001350520)	chr1:144868044	c.5881A>G (p.Ile1961Val)	0.232
14	SDK2 (NM_001144952)	chr17:71503692	c.109G>T (p.Val37Leu)	0.425
15	SDK2 (NM_001144952)	chr17:71503707	c.94C>G (p.Pro32Ala)	0.438
16	TENM3 (NM_001080477)	chr4:183658049	c.3056A>G (p.Lys1019Arg)	0.509
17	TSC2 (NM_000548)	chr16:2108740	c.849–8A>G	0.552

a WES and RNA sequencing was performed by the Illumina sequencing platform. The raw image data obtained was converted to raw sequence data by Base Calling software. The raw data are filtered and quality controlled to obtain clean data that meet the quality control requirements. The clean data were compared with the GRCh37/hg19 using Burrows-Wheeler Aligner (BWA) software. PCR duplicate fragments were removed using samblaster. Based on the comparison results, the sequencing depth, coverage, and comparison rate of the samples were counted.

**Figure 2:**
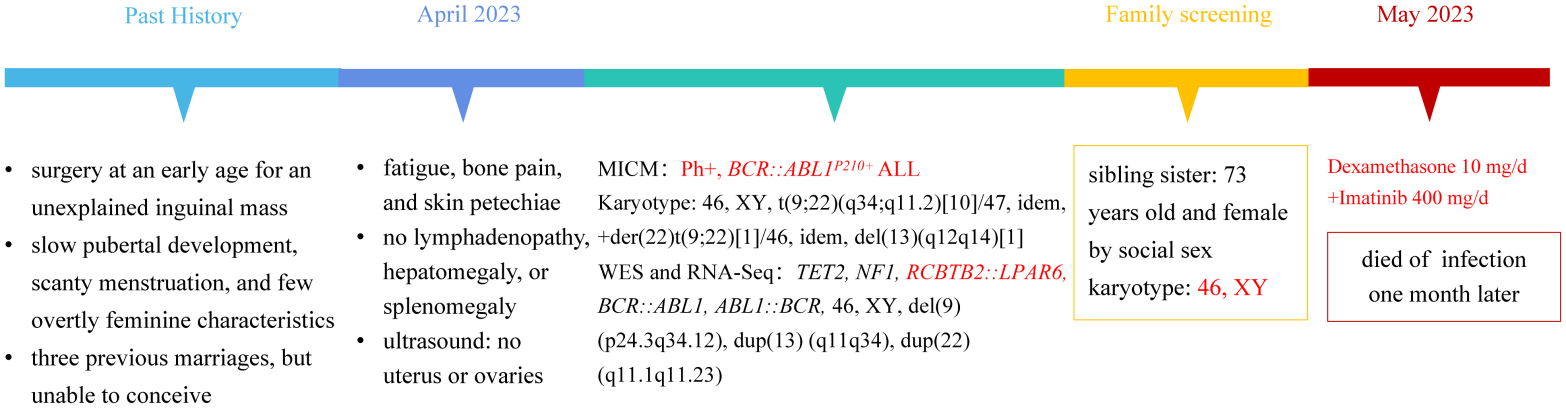
The patient’s treatment timeline.

We initially prescribed dexamethasone 10 mg/d to reduce the tumor load. Fortunately, the patient did not experience tumor lysis syndrome. The patient initially displayed doubt about the diagnosis, mistrust of the doctor, and refusal of family care. The patient had very low compliance and was agitated. After extensive deliberation and reassurance, the patient eventually consented to Imatinib 400 mg/d treatment. During the therapy, the patient developed pancytopenia combined with a severe lung infection, and ultimately died of the infection one month later. **Figure 2** shows the treatment timeline of this patient.

## Discussion

DSD is a congenital disorder that presents as incongruence in an individual’s sex chromosomes, gonads, and/or anatomical sex (4). Cases of 46, XY DSD are generally sporadic, and familial aggregation is extremely rare. Typically, 46, XY DSD patients will have varying degrees of masculinization of the external genitalia. Structures from Wolffian and Müllerian ducts also develop to varying degrees. The presence of testes can be detected by imaging in the majority of patients. Whereas 46, XY DSD can be identified through prenatal diagnosis, a portion is diagnosed at birth because of atypical external genitalia. In patients with more pronounced feminization, the probability of recognition at an early stage is lower, and most of them are diagnosed with 46, XY DSD by the presence of primary amenorrhea as well as unexplained inguinal hernia presenting in childhood, delayed breast development during puberty, or by the inability to be fertile. There are two main causes of 46, XY DSD, a decrease in the secretion of androgens (e.g., testosterone or dihydrotestosterone) during fetal sex differentiation, and impaired action of androgens on target tissues throughout the life course. The identification of novel genotypes has been made possible by WES. *WT1*, *GATA4*, *DHH*, *SOX9*, *NR5A1*, *MAP3K1*, *DHX37*, *and SRY* are the key potential genes that have been identified in 46, XY DSD. *DHCR7*, *STAR*, *CYP17*, *POR*, *B5*, *HSD3B2*, *HSD17B3*, and *CYP11A* are candidate genes for steroidogenic enzyme defects. Siblings sharing 46, XY DSD are very rare. We identified 11 pairs of siblings after synthesizing the published case reports (**Table 2**) (5–15). These patients were usually identified when they were young or in their teens, typically through observing vulvar anomalies or amenorrhea in the proband, followed by identifying comparable disorders in the siblings, leading to a certain diagnosis. Due to the lack of long-term follow-up, the development of hematologic disorders, especially hematologic malignancies, was not observed among these sibling pairs.

**Table 2:** Summary of case reports of siblings with 46, XY DSD.

Reference	Age at diagnose	Symptoms and physical examinations	Mutation etiology
Berg et al, 1987 (5)	20/18 years old	Primary amenorrhea, retarded breast development, scanty axillary and pubic hair growth.	gonadal dysgenesis
Shahid et al, 2007 (6)	23/27 years old	Primary amenorrhea, and clitoromegaly without labial fusion.	SRY mutations
Bisceglia et al, 2008 (7)	4 years old/ 3 months	Female external genitalia with a shortened vagina, normal for age breast development, absent pubic and axillary hair, no uterus, no fallopian tubes, and no ovaries.	complete androgen insensitivity
Nichols et al, 2008 (8)	17/17 years old	Primary amenorrhea, without appropriate breast and pubic hair development.	complete androgen insensitivity
Sharma et al, 2010 (9)	18^a^/12/8 years old	Axillary and pubic hair was scanty, with no hair growth, normal female genitalia, labia major, labia minor, and clitoris, with prominent bilateral inguinal swelling.	androgen insensitivity
Omrani et al, 2011 (10)	28/19/18 years old	Hirsutism and deepening of the voice, increased musculature, enlarged clitoris (6–7 cm), lack of breast development, and primary amenorrhea.	HSD17B3 mutations
Fabbri et al, 2014 (11)	S1: 9 months S2: at birth S3: at birth	S1: ambiguous genitalia S2: 2 cm phallus, penoscrotal hypospadias, and palpable gonads in the labioscrotal folds S3:1.3 cm phallus, a single perineal urogenital opening, and palpable gonads both in the inguinal region	NR5A1 mutations
Paris et al, 2017 (12)	26/23 years old	Primary amenorrhea, without breast development and scanty pubic hair.	homozygous DHH mutations
Banoth et al, 2017 (13)	29/23/17 years old	primary amenorrhea	gonadal dysgenesis
Ilaslan et al, 2018 (14)	S1: 17 years old S2: 8 months	S1: lack signs of puberty and menstruation, female-type external genitalia, oviducts, a normal prepubertal vagina, and uterus S2: ambiguous external genitalia (labioscrotum, perineal urethral orifice, and vaginal opening, phallic tubercle ca. 150 mm long)	gonadal dysgenesis, STARD8
Putri et al, 2022 (15)	19/17/15 years old	Late menarche, elementary breast development, and little axillary hair. The external genitals consisted of the vulva and major and minor labia. Clitoromegaly was present with a short (*<*5 cm) vagina. No female internal genital was found but undescended testes were palpable.	type 2 5-α reductase deficiency

a She did not have a vaginal opening, whereas the other siblings did. The urethral openings were normal in all three patients and no abnormal openings were seen. DHH: Desert hedgehog; HSD17B3, 17β-hydroxysteroid dehydrogenase type 3; NR5A1, Nuclear receptor subfamily 5 group A member 1; SRY, Sex-determining region Y; STARD8, STAR-related lipid transfer domain containing 8.

According to recent research (16–18), these patients are more likely than the general population to develop gonadal malignancies, such as germ cell tumors, embryonal carcinomas, yolk sac tumors, choriocarcinomas, and teratomas (19, 20). In contrast, the incidence of other cancers is not increased. The incidence of cardiovascular diseases like hypertension, atherosclerotic vascular disease, or coronary artery disease is not noticeably higher in patients with 46, XY DSD. Agnethe Berglund et al (21) retrospectively analyzed a total of 123 with 46, XY DSD in Denmark from 1960 to 2012. They had a higher incidence of congenital malformations and endocrine and gastrointestinal disorders. Five patients developed anemia. By the end of the follow-up, no hematologic malignancies were observed. Beyond that, there are no more large-sample data on survival and comorbidities, especially hematologic disorders in 46, XY DSD patients.

Because of the intricate pathophysiology of 46, XY DSD, and the vast range of clinical manifestations, therapeutic therapy has mostly focused on and battled with regulating the disorder’s classification. The incidence of hematologic malignancies in DSD patients is unknown. To ascertain whether there are differences in incidence, mutation type, and prognosis compared with the general population, extensive clinical data analysis is still needed. From an epidemiologic point of view, more data are needed to support whether initiating epidemiologic screening of patients with DSD has positive sociologic and medical significance. There are still many aspects of previous clinical reports of DSD patients that need to be improved. First, the number of cases is small, and the reports are mainly from single centers, which lack the integration of data from multiple centers. Secondly, there is a lack of long-term clinical follow-up, and there are very few studies on elderly DSD patients. Whether there is a difference in the incidence of tumors in this group of patients compared with the general population needs to be further confirmed.

Patients with 46, XY DSD are more likely to experience depression, obsessive-compulsive disorder, and an antisocial disposition (22). Self-harm and suicidal thoughts may emerge in a portion of patients (23, 24). The patient was admitted to the hospital with dangerously low platelets and extremely high white blood cell counts. Potential adverse consequences included hyperviscosity and resultant brain and gastrointestinal hemorrhage. The patient initially displayed doubt about the diagnosis, mistrust of the doctor, and refusal of family care when she was told that she was a 46, XY DSD patient and was diagnosed with Ph+ALL. The patient had very low compliance, was agitated, and rejected intravenous chemotherapy. After extensive deliberation and reassurance, the patient finally agreed to the regimen of intravenous dexamethasone pretreatment for lowering tumor load and imatinib maintenance therapy. The patient, who had coronary artery disease, type 2 diabetes, and hypertension, passed away during therapy as a result of an uncontrollable infection. The patient’s sister, on the other hand, had for some time denied our medical follow-up because she found it difficult to accept that she was a DSD patient and that her older sister’s condition was deteriorating so quickly. She finally accepted the diagnosis after extensive family and medical counseling, and she decided to stick with her decision to identify as female. The patient’s sister is currently compliant, and we will be following her closely. The focus of the follow-up will be the presence of cardiovascular events, endocrine disorders, and the occurrence of tumors.

Gender selection, identification, and the ideal decision-making window are the challenges in the management of 46, XY DSD. The major purpose of sex determination is to align sex determination with the chosen sex to avoid increased risk of sexual anxiety. After receiving a conclusive diagnosis, the patient should be engaged in a thorough and objective discussion about gender determination, including the likelihood of normal sexual function, fertility, risk of gonadal malignancy, and the various potential options. Advice should then be given in the patient’s specific situation. High-quality, long-term psychosocial evidence is scarce on gender identity in 46, XY DSD patients. The optimum path of treatment for addressing problems with gender identity needs active collaboration between the patient, their family, and the doctor.

## Conclusion

Studies of neoplasms in 46, XY DSD are limited to gonadal tumors, but the incidence and type of hematologic malignancies are incredibly uncommon. Siblings developing 46, XY DSD is extremely rare. We report the oldest patient diagnosed with 46, XY DSD. There have not been any reports of familial 46, XY DSD with a concurrent diagnosis of Ph+*BCR::ABL1^P210+^* ALL and a rarely reported *RCBTB2::LPAR6* fusion gene.

Nonetheless, given the scarcity of data on hematologic malignancies in DSD patients, widespread screening for hematologic abnormalities in this population is unlikely. What we can do is establish a database of patients with DSD with long-term follow-up to better monitor possible long-term comorbidities. Balancing the patient’s privacy and the social impact that long-term follow-up may have on the patient’s private life requires the cooperation of the patient’s family, the public, and the government.

## Data availability statement

The original contributions presented in the study are included in the article/supplementary material. Further inquiries can be directed to the corresponding authors.

## Ethics statement

The studies involving humans were approved by The First People’s Hospital of Yancheng. The studies were conducted in accordance with the local legislation and institutional requirements. The participants provided their written informed consent to participate in this study. Written informed consent was obtained from the individual(s) for the publication of any potentially identifiable images or data included in this article.

## Author contributions

LW: Conceptualization, Data curation, Formal analysis, Investigation, Methodology, Project administration, Resources, Software, Supervision, Validation, Visualization, Writing – original draft, Writing – review & editing. CX: Conceptualization, Data curation, Formal analysis, Investigation, Methodology, Project administration, Resources, Software, Supervision, Validation, Visualization, Writing – original draft, Writing – review & editing. XZ: Conceptualization, Data curation, Investigation, Methodology, Software, Writing – original draft. YH: Conceptualization, Formal analysis, Investigation, Project administration, Software, Validation, Writing – review & editing. HX: Formal analysis, Funding acquisition, Project administration, Resources, Validation, Visualization, Writing – review & editing. YM: Conceptualization, Data curation, Formal analysis, Investigation, Methodology, Project administration, Resources, Software, Supervision, Validation, Visualization, Writing – original draft, Writing – review & editing. YC: Conceptualization, Data curation, Formal analysis, Funding acquisition, Investigation, Methodology, Project administration, Resources, Software, Supervision, Validation, Visualization, Writing – original draft, Writing – review & editing.
